# Tetra­aqua­bis­[4-(4*H*-1,2,4-triazol-4-yl)benzoato-κ*N* ^1^]nickel(II) deca­hydrate

**DOI:** 10.1107/S1600536811051063

**Published:** 2011-12-03

**Authors:** Weixuan Sun, Yaqin Yu, Guanjun Wang, Xiaohui Wu

**Affiliations:** a Department of Preventive Medicine, School of Public Health, Jilin University, Changchun 130021, People’s Republic of China; b Department of Hematology, the First Hospital of Jilin University, Changchun 130021, People’s Republic of China; c School of Public Health, Jilin University, Changchun 130021, People’s Republic of China

## Abstract

In the title compound, [Ni(C_9_H_6_N_3_O_2_)_2_(H_2_O)_4_]·10H_2_O, the Ni^II^ ion lies on a twofold rotation axis and displays a slightly distorted octa­hedral geometry defined by two N atoms from two monodentate 4-(1,2,4-triazol-4-yl)benzoate ligands and four water mol­ecules, two of which also lie on the twofold rotation axis. In the crystal, the complex mol­ecules and uncoordinated water mol­ecules are linked *via* inter­molecular O—H⋯N and O—H⋯O hydrogen bonds, forming a three-dimensional supra­molecular network. π–π inter­actions between the benzene rings provide additional stability of the crystal packing [centroid–centroid distance = 3.792 (2) Å].

## Related literature

For general background to the applications and structures of metal coordination polymers, see: Rowsell & Yaghi (2005 ▶); Su *et al.* (2010 ▶); Wang *et al.* (2009 ▶); Zhang & Chen (2008 ▶). For a related structure, see: Cui & Zhao (2011 ▶).
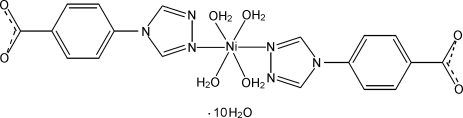


## Experimental

### 

#### Crystal data

[Ni(C_9_H_6_N_3_O_2_)_2_(H_2_O)_4_]·10H_2_O
*M* *_r_* = 687.25
Monoclinic, 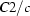

*a* = 25.840 (3) Å
*b* = 7.8664 (8) Å
*c* = 16.8013 (17) Å
β = 112.712 (1)°
*V* = 3150.3 (6) Å^3^
*Z* = 4
Mo *K*α radiation
μ = 0.70 mm^−1^
*T* = 293 K
0.22 × 0.20 × 0.19 mm

#### Data collection

Bruker APEXII CCD diffractometer
Absorption correction: multi-scan (*SADABS*; Bruker, 2001 ▶) *T*_min_ = 0.83, *T*_max_ = 0.90
8290 measured reflections
3079 independent reflections
2273 reflections with *I* > 2σ(*I*)
*R*_int_ = 0.057

#### Refinement

*R*[*F* ^2^ > 2σ(*F* ^2^)] = 0.049
*wR*(*F* ^2^) = 0.119
*S* = 1.04
3079 reflections
238 parameters
14 restraints
H atoms treated by a mixture of independent and constrained refinement
Δρ_max_ = 0.55 e Å^−3^
Δρ_min_ = −0.63 e Å^−3^

### 

Data collection: *APEX2* (Bruker, 2007 ▶); cell refinement: *SAINT* (Bruker, 2007 ▶); data reduction: *SAINT*; program(s) used to solve structure: *SHELXTL* (Sheldrick, 2008 ▶); program(s) used to refine structure: *SHELXTL*; molecular graphics: *SHELXTL*; software used to prepare material for publication: *SHELXTL*.

## Supplementary Material

Crystal structure: contains datablock(s) global, I. DOI: 10.1107/S1600536811051063/hy2491sup1.cif

Structure factors: contains datablock(s) I. DOI: 10.1107/S1600536811051063/hy2491Isup2.hkl

Additional supplementary materials:  crystallographic information; 3D view; checkCIF report

## Figures and Tables

**Table 1:** Hydrogen-bond geometry (Å, °)

*D*—H⋯*A*	*D*—H	H⋯*A*	*D*⋯*A*	*D*—H⋯*A*
O3—H3*A*⋯O1^i^	0.84 (2)	1.93 (2)	2.751 (3)	167 (4)
O4—H4*A*⋯O1^ii^	0.84 (2)	1.86 (2)	2.692 (3)	172 (4)
O5—H5*A*⋯O8	0.82 (2)	1.94 (2)	2.752 (3)	169 (4)
O5—H5*B*⋯O7	0.83 (2)	1.84 (2)	2.670 (3)	171 (4)
O6—H6*A*⋯O7	0.82 (2)	1.95 (2)	2.773 (4)	178 (4)
O6—H6*B*⋯O10	0.84 (2)	1.91 (3)	2.747 (4)	177 (6)
O7—H7*A*⋯O2^iii^	0.84 (2)	1.84 (2)	2.674 (3)	170 (4)
O7—H7*B*⋯O9^iv^	0.84 (2)	1.88 (2)	2.715 (4)	171 (4)
O8—H8*A*⋯N3^v^	0.82 (2)	2.16 (2)	2.943 (3)	160 (4)
O8—H8*B*⋯O2^vi^	0.85 (2)	1.92 (2)	2.763 (3)	175 (4)
O9—H9*A*⋯O1^ii^	0.84 (2)	1.93 (2)	2.751 (3)	169 (4)
O9—H9*B*⋯O8	0.85 (2)	1.93 (2)	2.757 (3)	164 (4)

Symmetry codes: (i) 
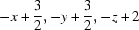
; (ii) 
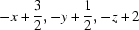
; (iii) 
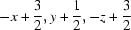
; (iv) 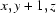; (v) 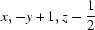; (vi) 
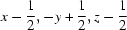
.

**Fig. 1.**
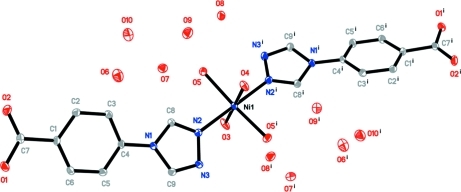
The molecular structute of the title compound. Displacement ellipsoids are drawn at the 30% probability level. H atoms have been omitted for clarity. [Symmetry code: (i) 1-x, y, 3/2-z.]

**Fig. 2.**
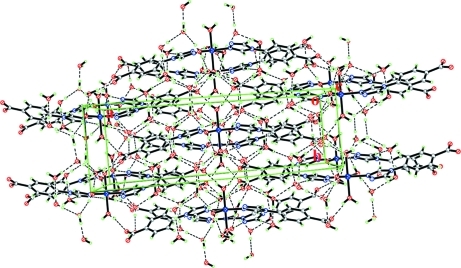
View of the three-dimensional structure of the title compound built by hydrogen bonds (dashed lines).

## supplementary crystallographic information

### Comment

Recently, the chemists have devoted themselves to design and synthesize coordination polymers, not only due to their potential applications in the realm of gas adsorption and separation, catalysis, magnetism, luminescence, host–guest chemistry *etc*, but also for their aesthetic and often complicated architectures and topologies (Su *et al.*, 2010; Wang *et al.*, 2009). It is well known that carboxylic acids are excellent building blocks for the construction of coordination polymers because the carboxylate groups may induce core aggregation and link these discrete clusters into an extended framework by virtue of its bridging ability (Rowsell & Yaghi, 2005; Zhang & Chen, 2008). Taking these into account, we chose a carboxylate ligand, 4-(1,2,4-triazol-4-yl)benzoic acid, generating the title compound, which is reported here.

In the title compound, the Ni^II^ ion lies on a twofold rotation axis and displays a slightly distorted octahedral geometry defined by two N atoms from two 4-(1,2,4-triazol-4-yl)benzoate ligands and four water molecules, two of which lie on the twofold rotation axis (Fig. 1). The bond lengths and angles are in a normal range (Cui & Zhao, 2011). In the crystal, the complex molecules and uncoordinated water molecules are linked *via* intermolecular O—H···N and O—H···O hydrogen bonds, forming a three-dimensional supramolecular network (Fig. 2). π–π interactions between the benzene rings, with a centroid–centroid distance of 3.792 (2) Å, provide additional stability of the crystal packing.

### Experimental

The synthesis was performed under hydrothermal conditions. A mixture of Ni(CH_3_COO)_2_.4H_2_O (0.2 mmol, 0.050 g), 4-(1,2,4-triazol-4-yl)benzoic acid (0.4 mmol, 0.075 g), NaOH (0.4 mmol, 0.016 g) and H_2_O (15 ml) in a 25 ml stainless steel reactor with a Teflon liner was heated from 293 to 443 K in 2 h and a constant temperature was maintained at 443 K for 72 h. After the mixture was cooled to 298 K, pink crystals of the title compound were obtained from the reaction.

### Refinement

H atoms on C atoms were positioned geometrically and refined as riding atoms, with C—H = 0.93 Å and with *U*_iso_(H) = 1.2*U*_eq_(C). H atoms bonded to O atoms were located in a difference Fourier map and refined with O—H distance restraints of 0.85 (2) Å and with *U*_iso_(H) = 0.054 Å^2^.

### Crystal data

**Table:**

[Ni(C_9_H_6_N_3_O_2_)_2_(H_2_O)_4_]·10H_2_O	*F*(000) = 1448
*M**_r_* = 687.25	*D*_x_ = 1.449 Mg m^−^^3^
Monoclinic, *C*2/*c*	Mo *K*α radiation, λ = 0.71073 Å
Hall symbol: -C 2yc	Cell parameters from 3079 reflections
*a* = 25.840 (3) Å	θ = 1.0–25.9°
*b* = 7.8664 (8) Å	µ = 0.70 mm^−^^1^
*c* = 16.8013 (17) Å	*T* = 293 K
β = 112.712 (1)°	Block, pink
*V* = 3150.3 (6) Å^3^	0.22 × 0.20 × 0.19 mm
*Z* = 4	

### Data collection

**Table:**

Bruker APEXII CCD diffractometer	3079 independent reflections
Radiation source: fine-focus sealed tube	2273 reflections with *I* > 2σ(*I*)
graphite	*R*_int_ = 0.057
φ and ω scans	θ_max_ = 25.9°, θ_min_ = 2.5°
Absorption correction: multi-scan (*SADABS*; Bruker, 2001)	*h* = −31→31
*T*_min_ = 0.83, *T*_max_ = 0.90	*k* = −6→9
8290 measured reflections	*l* = −20→17

### Refinement

**Table:**

Refinement on *F*^2^	Primary atom site location: structure-invariant direct methods
Least-squares matrix: full	Secondary atom site location: difference Fourier map
*R*[*F*^2^ > 2σ(*F*^2^)] = 0.049	Hydrogen site location: inferred from neighbouring sites
*wR*(*F*^2^) = 0.119	H atoms treated by a mixture of independent and constrained refinement
*S* = 1.04	*w* = 1/[σ^2^(*F*_o_^2^) + (0.0451*P*)^2^ + 2.4003*P*] where *P* = (*F*_o_^2^ + 2*F*_c_^2^)/3
3079 reflections	(Δ/σ)_max_ < 0.001
238 parameters	Δρ_max_ = 0.55 e Å^−^^3^
14 restraints	Δρ_min_ = −0.63 e Å^−^^3^

### Fractional atomic coordinates and isotropic or equivalent isotropic displacement parameters (Å^2^)

**Table:**

	*x*	*y*	*z*	*U*_iso_*/*U*_eq_	
Ni1	0.5000	0.59822 (8)	0.7500	0.01918 (19)	
C1	0.83480 (12)	0.4306 (4)	1.04342 (19)	0.0186 (7)	
C2	0.79863 (12)	0.3835 (4)	0.96082 (19)	0.0200 (7)	
H2	0.8126	0.3236	0.9257	0.024*	
C3	0.74225 (13)	0.4242 (4)	0.93015 (19)	0.0205 (7)	
H3	0.7182	0.3890	0.8755	0.025*	
C4	0.72201 (12)	0.5180 (4)	0.98169 (19)	0.0163 (7)	
C5	0.75769 (13)	0.5702 (4)	1.0635 (2)	0.0209 (7)	
H5	0.7441	0.6351	1.0975	0.025*	
C6	0.81360 (12)	0.5246 (4)	1.0939 (2)	0.0194 (7)	
H6	0.8374	0.5575	1.1491	0.023*	
C7	0.89538 (13)	0.3748 (4)	1.0778 (2)	0.0196 (7)	
C8	0.62534 (12)	0.5537 (4)	0.86818 (19)	0.0192 (7)	
H8	0.6334	0.5222	0.8209	0.023*	
C9	0.63313 (13)	0.6112 (4)	0.9971 (2)	0.0231 (7)	
H9	0.6480	0.6275	1.0565	0.028*	
N1	0.66358 (10)	0.5587 (3)	0.95053 (15)	0.0170 (6)	
N2	0.57580 (10)	0.5986 (3)	0.86385 (15)	0.0193 (6)	
N3	0.58082 (11)	0.6353 (4)	0.94733 (16)	0.0230 (7)	
O1	0.92384 (9)	0.3974 (3)	1.15821 (13)	0.0218 (5)	
O2	0.91424 (9)	0.3070 (3)	1.02773 (14)	0.0302 (6)	
O3	0.5000	0.8594 (4)	0.7500	0.0294 (8)	
O4	0.5000	0.3349 (4)	0.7500	0.0228 (7)	
O5	0.54970 (9)	0.5800 (3)	0.67831 (14)	0.0211 (5)	
O6	0.70477 (12)	0.6665 (4)	0.74921 (19)	0.0430 (7)	
O7	0.60752 (10)	0.8271 (3)	0.64103 (15)	0.0280 (6)	
O8	0.52147 (10)	0.3127 (3)	0.56372 (15)	0.0255 (6)	
O9	0.60244 (10)	0.1461 (3)	0.69923 (16)	0.0315 (6)	
O10	0.69525 (11)	0.3274 (4)	0.70686 (17)	0.0363 (7)	
H3A	0.5254 (13)	0.921 (4)	0.784 (2)	0.054*	
H4A	0.5231 (14)	0.265 (4)	0.783 (2)	0.054*	
H5A	0.5411 (17)	0.510 (4)	0.639 (2)	0.054*	
H5B	0.5653 (16)	0.656 (4)	0.661 (3)	0.054*	
H6A	0.6758 (12)	0.715 (5)	0.718 (2)	0.054*	
H6B	0.7026 (18)	0.562 (3)	0.738 (3)	0.054*	
H7A	0.6022 (17)	0.833 (5)	0.5885 (14)	0.054*	
H7B	0.6041 (18)	0.929 (3)	0.654 (3)	0.054*	
H8A	0.5304 (17)	0.319 (6)	0.5218 (19)	0.054*	
H8B	0.4879 (10)	0.279 (5)	0.550 (3)	0.054*	
H9A	0.5962 (17)	0.146 (6)	0.7445 (18)	0.054*	
H9B	0.5732 (12)	0.191 (5)	0.662 (2)	0.054*	
H10A	0.7237 (12)	0.268 (5)	0.727 (2)	0.054*	
H10B	0.6695 (14)	0.275 (5)	0.712 (3)	0.054*	

### Atomic displacement parameters (Å^2^)

**Table:**

	*U*^11^	*U*^22^	*U*^33^	*U*^12^	*U*^13^	*U*^23^
Ni1	0.0147 (3)	0.0216 (3)	0.0196 (3)	0.000	0.0049 (2)	0.000
C1	0.0117 (15)	0.0238 (18)	0.0189 (16)	0.0005 (13)	0.0044 (13)	0.0017 (14)
C2	0.0179 (17)	0.0281 (19)	0.0144 (16)	0.0013 (14)	0.0069 (13)	−0.0005 (14)
C3	0.0155 (16)	0.0305 (19)	0.0121 (15)	−0.0005 (14)	0.0014 (12)	0.0006 (14)
C4	0.0105 (15)	0.0197 (16)	0.0172 (16)	0.0021 (13)	0.0035 (13)	0.0023 (14)
C5	0.0188 (17)	0.0258 (19)	0.0174 (16)	0.0041 (14)	0.0062 (13)	−0.0042 (14)
C6	0.0153 (16)	0.0223 (17)	0.0170 (16)	−0.0007 (13)	0.0024 (13)	−0.0029 (14)
C7	0.0145 (16)	0.0217 (18)	0.0214 (17)	0.0004 (13)	0.0057 (14)	0.0031 (15)
C8	0.0144 (16)	0.0263 (18)	0.0148 (15)	0.0000 (13)	0.0034 (13)	−0.0012 (14)
C9	0.0170 (17)	0.037 (2)	0.0146 (16)	0.0034 (15)	0.0055 (13)	−0.0029 (15)
N1	0.0123 (13)	0.0229 (15)	0.0136 (13)	0.0014 (11)	0.0026 (10)	−0.0014 (11)
N2	0.0151 (13)	0.0272 (15)	0.0155 (13)	−0.0008 (12)	0.0058 (11)	−0.0021 (12)
N3	0.0144 (14)	0.0370 (17)	0.0149 (14)	0.0017 (12)	0.0026 (11)	−0.0043 (13)
O1	0.0167 (11)	0.0254 (12)	0.0180 (11)	0.0023 (10)	0.0010 (9)	0.0010 (10)
O2	0.0169 (13)	0.0498 (17)	0.0221 (13)	0.0123 (12)	0.0056 (10)	0.0020 (12)
O3	0.0210 (19)	0.0179 (18)	0.032 (2)	0.000	−0.0087 (15)	0.000
O4	0.0194 (18)	0.0150 (17)	0.0248 (19)	0.000	−0.0015 (14)	0.000
O5	0.0195 (12)	0.0248 (13)	0.0213 (12)	−0.0055 (10)	0.0106 (10)	−0.0034 (10)
O6	0.0341 (17)	0.0381 (17)	0.0489 (18)	0.0012 (14)	0.0073 (14)	0.0007 (15)
O7	0.0318 (14)	0.0289 (14)	0.0249 (13)	−0.0017 (12)	0.0126 (12)	0.0015 (12)
O8	0.0190 (13)	0.0378 (15)	0.0209 (12)	−0.0077 (11)	0.0090 (10)	−0.0023 (11)
O9	0.0255 (14)	0.0388 (15)	0.0315 (15)	0.0064 (12)	0.0123 (12)	0.0047 (13)
O10	0.0282 (16)	0.0390 (17)	0.0373 (16)	−0.0016 (12)	0.0080 (13)	0.0061 (13)

### Geometric parameters (Å, °)

**Table:**

Ni1—O3	2.054 (3)	C8—H8	0.9300
Ni1—O4	2.071 (3)	C9—N3	1.300 (4)
Ni1—O5	2.077 (2)	C9—N1	1.370 (4)
Ni1—N2	2.145 (2)	C9—H9	0.9300
C1—C6	1.387 (4)	N2—N3	1.388 (3)
C1—C2	1.391 (4)	O3—H3A	0.836 (18)
C1—C7	1.510 (4)	O4—H4A	0.840 (18)
C2—C3	1.382 (4)	O5—H5A	0.822 (19)
C2—H2	0.9300	O5—H5B	0.835 (19)
C3—C4	1.385 (4)	O6—H6A	0.824 (19)
C3—H3	0.9300	O6—H6B	0.838 (19)
C4—C5	1.389 (4)	O7—H7A	0.840 (18)
C4—N1	1.430 (4)	O7—H7B	0.844 (19)
C5—C6	1.381 (4)	O8—H8A	0.823 (18)
C5—H5	0.9300	O8—H8B	0.848 (19)
C6—H6	0.9300	O9—H9A	0.836 (19)
C7—O2	1.243 (4)	O9—H9B	0.847 (19)
C7—O1	1.277 (4)	O10—H10A	0.824 (19)
C8—N2	1.303 (4)	O10—H10B	0.817 (19)
C8—N1	1.355 (4)		
			
O3—Ni1—O4	180.000 (2)	C5—C6—C1	121.1 (3)
O3—Ni1—O5^i^	93.97 (6)	C5—C6—H6	119.5
O4—Ni1—O5^i^	86.03 (7)	C1—C6—H6	119.5
O3—Ni1—O5	93.97 (7)	O2—C7—O1	124.0 (3)
O4—Ni1—O5	86.03 (7)	O2—C7—C1	119.0 (3)
O5^i^—Ni1—O5	172.07 (13)	O1—C7—C1	116.9 (3)
O3—Ni1—N2	89.93 (7)	N2—C8—N1	111.3 (3)
O4—Ni1—N2	90.07 (7)	N2—C8—H8	124.4
O5^i^—Ni1—N2	92.24 (9)	N1—C8—H8	124.4
O5—Ni1—N2	87.77 (9)	N3—C9—N1	111.2 (3)
O3—Ni1—N2^i^	89.93 (7)	N3—C9—H9	124.4
O4—Ni1—N2^i^	90.07 (7)	N1—C9—H9	124.4
O5^i^—Ni1—N2^i^	87.77 (9)	C8—N1—C9	103.8 (3)
O5—Ni1—N2^i^	92.24 (9)	C8—N1—C4	128.2 (3)
N2—Ni1—N2^i^	179.85 (16)	C9—N1—C4	128.1 (2)
C6—C1—C2	118.7 (3)	C8—N2—N3	107.1 (2)
C6—C1—C7	121.1 (3)	C8—N2—Ni1	126.1 (2)
C2—C1—C7	120.1 (3)	N3—N2—Ni1	126.73 (18)
C3—C2—C1	121.0 (3)	C9—N3—N2	106.7 (2)
C3—C2—H2	119.5	Ni1—O3—H3A	125 (3)
C1—C2—H2	119.5	Ni1—O4—H4A	131 (3)
C2—C3—C4	119.3 (3)	Ni1—O5—H5A	118 (3)
C2—C3—H3	120.4	Ni1—O5—H5B	130 (3)
C4—C3—H3	120.4	H5A—O5—H5B	103 (4)
C3—C4—C5	120.6 (3)	H6A—O6—H6B	110 (4)
C3—C4—N1	119.4 (3)	H7A—O7—H7B	103 (4)
C5—C4—N1	120.0 (3)	H8A—O8—H8B	112 (4)
C6—C5—C4	119.3 (3)	H9A—O9—H9B	103 (4)
C6—C5—H5	120.4	H10A—O10—H10B	108 (4)
C4—C5—H5	120.4		
			
C6—C1—C2—C3	−2.1 (5)	C3—C4—N1—C8	−17.2 (5)
C7—C1—C2—C3	175.9 (3)	C5—C4—N1—C8	163.8 (3)
C1—C2—C3—C4	2.0 (5)	C3—C4—N1—C9	162.5 (3)
C2—C3—C4—C5	−0.3 (5)	C5—C4—N1—C9	−16.5 (5)
C2—C3—C4—N1	−179.3 (3)	N1—C8—N2—N3	−0.1 (4)
C3—C4—C5—C6	−1.3 (5)	N1—C8—N2—Ni1	−176.2 (2)
N1—C4—C5—C6	177.7 (3)	O3—Ni1—N2—C8	−109.7 (3)
C4—C5—C6—C1	1.2 (5)	O4—Ni1—N2—C8	70.3 (3)
C2—C1—C6—C5	0.4 (5)	O5^i^—Ni1—N2—C8	156.4 (3)
C7—C1—C6—C5	−177.5 (3)	O5—Ni1—N2—C8	−15.7 (3)
C6—C1—C7—O2	−172.3 (3)	O3—Ni1—N2—N3	75.0 (2)
C2—C1—C7—O2	9.8 (5)	O4—Ni1—N2—N3	−105.0 (2)
C6—C1—C7—O1	9.3 (5)	O5^i^—Ni1—N2—N3	−19.0 (3)
C2—C1—C7—O1	−168.6 (3)	O5—Ni1—N2—N3	169.0 (3)
N2—C8—N1—C9	−0.1 (4)	N1—C9—N3—N2	−0.4 (4)
N2—C8—N1—C4	179.7 (3)	C8—N2—N3—C9	0.3 (4)
N3—C9—N1—C8	0.3 (4)	Ni1—N2—N3—C9	176.4 (2)
N3—C9—N1—C4	−179.5 (3)		

Symmetry codes: (i) −*x*+1, *y*, −*z*+3/2.

### Hydrogen-bond geometry (Å, °)

**Table:**

*D*—H···*A*	*D*—H	H···*A*	*D*···*A*	*D*—H···*A*
O3—H3A···O1^ii^	0.84 (2)	1.93 (2)	2.751 (3)	167 (4)
O4—H4A···O1^iii^	0.84 (2)	1.86 (2)	2.692 (3)	172 (4)
O5—H5A···O8	0.82 (2)	1.94 (2)	2.752 (3)	169 (4)
O5—H5B···O7	0.83 (2)	1.84 (2)	2.670 (3)	171 (4)
O6—H6A···O7	0.82 (2)	1.95 (2)	2.773 (4)	178 (4)
O6—H6B···O10	0.84 (2)	1.91 (3)	2.747 (4)	177 (6)
O7—H7A···O2^iv^	0.84 (2)	1.84 (2)	2.674 (3)	170 (4)
O7—H7B···O9^v^	0.84 (2)	1.88 (2)	2.715 (4)	171 (4)
O8—H8A···N3^vi^	0.82 (2)	2.16 (2)	2.943 (3)	160 (4)
O8—H8B···O2^vii^	0.85 (2)	1.92 (2)	2.763 (3)	175 (4)
O9—H9A···O1^iii^	0.84 (2)	1.93 (2)	2.751 (3)	169 (4)
O9—H9B···O8	0.85 (2)	1.93 (2)	2.757 (3)	164 (4)

Symmetry codes: (ii) −*x*+3/2, −*y*+3/2, −*z*+2; (iii) −*x*+3/2, −*y*+1/2, −*z*+2; (iv) −*x*+3/2, *y*+1/2, −*z*+3/2; (v) *x*, *y*+1, *z*; (vi) *x*, −*y*+1, *z*−1/2; (vii) *x*−1/2, −*y*+1/2, *z*−1/2.
